# Antibacterial Effects of Glycyrrhetinic Acid and Its Derivatives on *Staphylococcus aureus*

**DOI:** 10.1371/journal.pone.0165831

**Published:** 2016-11-07

**Authors:** Kentaro Oyama, Miki Kawada-Matsuo, Yuichi Oogai, Tetsuya Hayashi, Norifumi Nakamura, Hitoshi Komatsuzawa

**Affiliations:** 1 Department of Oral Microbiology, Kagoshima University Graduate School of Medical and Dental Sciences, Kagoshima, Japan; 2 Department of Oral maxillofacial Surgery, Kagoshima University Graduate School of Medical and Dental Sciences, Kagoshima, Japan; 3 Department of Bacteriology, Faculty of Medical Sciences, Kyushu University, Fukuoka, Japan; University Medical Center Utrecht, NETHERLANDS

## Abstract

*Staphylococcus aureus* is a major pathogen in humans and causes serious problems due to antibiotic resistance. We investigated the antimicrobial effect of glycyrrhetinic acid (GRA) and its derivatives against 50 clinical *S*. *aureus* strains, including 18 methicillin-resistant strains. The minimum inhibitory concentrations (MICs) of GRA, dipotassium glycyrrhizate, disodium succinoyl glycyrrhetinate (GR-SU), stearyl glycyrrhetinate and glycyrrhetinyl stearate were evaluated against various *S*. *aureus* strains. Additionally, we investigated the bactericidal effects of GRA and GR-SU against two specific *S*. *aureus* strains. DNA microarray analysis was also performed to clarify the mechanism underlying the antibacterial activity of GR-SU. We detected the antimicrobial activities of five agents against *S*. *aureus* strains. GRA and GR-SU showed strong antibacterial activities compared to the other three agents tested. At a higher concentration (above 2x MIC), GRA and GR-SU showed bactericidal activity, whereas at a concentration of 1x MIC, they showed a bacteriostatic effect. Additionally, GRA and GR-SU exhibited a synergistic effect with gentamicin. The expression of a large number of genes (including transporters) and metabolic factors (carbohydrates and amino acids) was altered by the addition of GR-SU, suggesting that the inhibition of these metabolic processes may influence the degree of the requirement for carbohydrates or amino acids. In fact, the requirement for carbohydrates or amino acids was increased in the presence of either GRA or GR-SU. GRA and GR-SU exhibited strong antibacterial activity against several *S*. *aureus* strains, including MRSA. This activity may be partly due to the inhibition of several pathways involved in carbohydrate and amino acid metabolism.

## Introduction

*Staphylococcus aureus* is a commensal bacterium in humans that can also be pathogenic primarily as an opportunistic infectious agent. *S*. *aureus* causes various suppurative diseases, food poisoning, pneumonia and sepsis [1, 2, 3]. Chemotherapeutic treatment is generally applied to *S*. *aureus* infectious diseases. However, the emergence of methicillin-resistant *Staphylococcus aureus* (MRSA) represents a serious problem for the treatment of *S*. *aureus* infections. Since most clinical MRSA strains exhibit a high level of multidrug resistance, the treatment of MRSA infections is now a serious medical concern worldwide [4, 5].

Glycopeptides such as vancomycin and teicoplanin are often used to treat MRSA infections [6]. However, vancomycin-intermediate resistant *S*. *aureus* (VISA) emerged in the late 1990s [7, 8]. Furthermore, vancomycin-resistant *S*. *aureus* (VRSA) was first reported in Michigan in 2007 [9]. Daptomycin, which is an antibacterial agent against MRSA, has been used recently, but daptomycin-resistant strains have also been reported [10]. Thus, it is very likely that chemotherapy against *S*. *aureus* infections will become more difficult in the future.

Some plant extracts have long been known to exert antibacterial effects. Among these, polyphenols, which are classified into flavonoids (e.g., catechin, flavonol, and tannin) and nonflavonoids (e.g., phenolic acid, neolignan), have been especially well studied [11, 12, 13, 14]. For example, it has been shown that tannins from tea leaves or persimmon have antibacterial effects against *Streptococcus mutans*, *S*. *aureus* and *Escherichia coli*. [15, 16]. Polyphenols are thought to be less likely to induce the emergence of resistant strains than general antibiotics because the mode of action of polyphenols has been demonstrated to cause cytoplasmic membrane destabilization and/or permeabilization [11]. The side effects of these agents are also thought to be less severe than those of traditional antibiotics.

Licorice is a Leguminosae perennial found in the Mediterranean region, south Russia, central Asia, northern China and America. This plant has been traditionally used as a medicinal agent due to its antibacterial, anti-viral, anti-inflammatory and calming effects [17–20]. Glycyrrhetinic acid is a major component in licorice extract. Previous studies have shown that glycyrrhetinic acid has many properties, including anti-inflammatory, anti-allergic, anti-peptic ulcer, and anti-viral activities [21–25]. Its anti-inflammatory effect is particularly well known and has been applied to clinical therapy. It has been reported that the structure of glycyrrhetinic acid is similar to that of cortisone, a steroid hormone that exerts a strong anti-inflammatory effect [23]. In Japan, a preparation of monoammonium glycyrrhizinate (Stronger Neo-Minophagen C) is used to treat chronic hepatitis [26, 27]. Glycyrrhetinic acid has also been reported to have antibacterial effects against some bacterial species, but the mechanism underlying this activity remains unknown [28, 29]. Additionally, several derivatives of glycyrrhetinic acid exhibit anti-inflammatory activities, but their antibacterial activities have not been elucidated [30, 31, 32].

The aims of this study are to evaluate the antibacterial effects of glycyrrhetinic acid and its derivatives against clinical *S*. *aureus* strains and to investigate the mechanism underlying their antibacterial effect against *S*. *aureus*.

## Materials and Methods

### Bacterial strains and culture

*S*. *aureus* was cultured in tryptic soy broth (TSB) at 37°C with shaking at 50 rpm. Fifty clinical *S*. *aureus* strains, including 31 methicillin-sensitive *S*. *aureus* (MSSA) strains and 19 MRSA strains, were used in this study (S1 Table). These strains were from laboratory collection of *S*. *aureus* clinical isolates at Department of Bacteriology, Hiroshima University Graduate School of Biomedical and Health Sciences. A chemically defined medium (CDM) supplemented with glucose (50 mM) as the sole carbon source was prepared [33]. When necessary, glucose was replaced with other sugars (lactose, trehalose, and sucrose).

### Glycyrrhetinic acid derivatives

**Fig 1:**
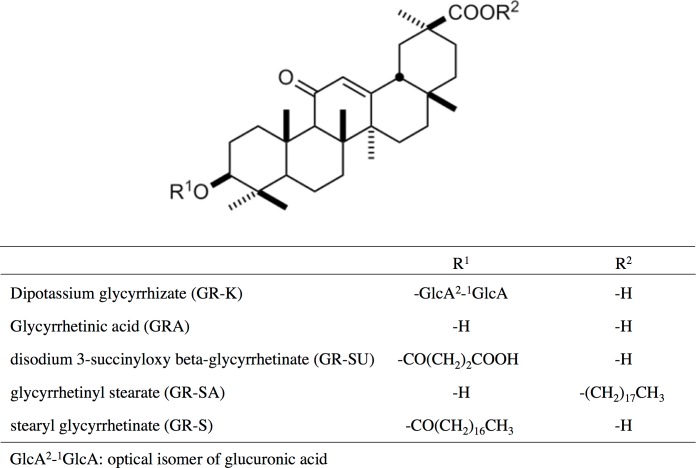
Structures of GRA and its derivatives.

Glycyrrhetinic acid (GRA) and its derivatives used in this study are shown in Fig 1. These agents were obtained from Maruzen Pharmaceuticals Co., Ltd., Hiroshima, Japan. Dipotassium glycyrrhizate (GR-K) and disodium succinoyl glycyrrhetinate (GR-SU) were solubilized in distilled water. Glycyrrhetinic acid (GRA) was solubilized in 100% dimethyl sulfoxide (DMSO). Stearyl glycyrrhetinate (GR-S) and glycyrrhetinyl stearate (GR-SA) were solubilized in 100% ethanol. Stock solutions were prepared at a concentration of 20 mg/ml and were diluted in medium to the appropriate concentrations indicated in each experiment.

### Determination of the minimum inhibitory concentration (MIC)

The MICs were determined by using the micro-dilution method as previously described [33]. Briefly, each GRA derivative was adjusted to 4,096 mg/L in TSB, and 2-fold serial dilutions were prepared in a 96-well microplate (Thermo Fisher Scientific, Roskilde, Denmark). Overnight bacterial cultures were adjusted to an OD_660_ of 1.0 (1x10^9^ cells/ml) and diluted to 1:100 with TSB (1x10^7^ cells/ml). Ten microliters of the bacterial culture (1x10^5^ cells/well) was applied to each well (100 μl total volume). The MICs of glycyrrhetinic acid and its derivatives were determined after the plate was incubated for 24 h at 37°C.

### *S*. *aureus* growth curve

Overnight cultures of *S*. *aureus* MW2 were adjusted to an OD_660_ of 1.0. Then, 100 μl of bacterial culture was inoculated into 5 ml of TSB and incubated at 37°C with shaking. When the OD_660_ reached 0.3, various concentrations of either GRA or GR-SU were added to the medium, and the growth was monitored. To investigate cell viability, appropriate dilutions of TSB after the addition of either GRA or GR-SU were inoculated onto a tryptic soy agar (TSA) plate. After overnight incubation at 37°C, the colony forming units (CFUs) were counted. Three independent experiments were performed, and the mean ± SD was calculated. The data were analyzed for statistically significant differences compared to untreated control at each timepoint by a two-way ANOVA followed by Dunnett’s post hoc test.

### Effects of GRA and GR-SU on cell viability

Overnight cultures of *S*. *aureus* MW2 cells were washed with 10 mM sodium phosphate buffer (PB; pH 6.8) and suspended in PB. The bacterial suspension was diluted to 10^7^ cells/ml with PB, and 10 μl of the diluted suspension was inoculated into 500 μl of PB containing various concentrations of GR-SU (1x MIC: 128 mg/L) or GRA (1x MIC: 512 mg/L) and incubated for 1 or 2 hours at 37°C. Dilutions of the reaction mixture (100 μl) were plated onto TSA plates and incubated at 37°C overnight. The antibacterial effect was calculated as the ratio of the number of surviving cells (survival rate %) to the total number of bacteria incubated in PB. To verify the cell number of each strain inoculated in PB, the dilutions of the bacterial suspension prior to inoculation were plated onto TSA and incubated at 37°C overnight. Three independent experiments were performed, and the mean ± SD was calculated. The data were analyzed for significant differences compared to untreated control using two-way ANOVA followed by Dunnett’s post hoc test.

### Effects of GRA and GR-SU in combination with antibiotics

The effects of combination treatments of gentamicin (Wako Pure Chemical Industries Ltd, Osaka, Japan), chloramphenicol (Sigma-Aldrich Corporation, Tokyo, Japan), tetracycline (Wako Pure Chemical Industries Ltd), oxacillin (Sigma-Aldrich Corporation) or ofloxacin (Daiichi Sankyo Company, Tokyo, Japan) with either GRA or GR-SU were investigated. Overnight cultures of *S*. *aureus* MW2 were adjusted to 10^7^ cells/ml, and 10 μL of the bacterial suspension was applied to each well in a 96-well plate. Antibiotics were dissolved in TSB alone or TSB containing either GRA or GR-SU (1/2, 1/4 or 1/8 MICs for each agent). Stock solutions (1 g/L) of gentamicin, oxacillin or ofloxacin were prepared in TSB or TSB containing either GRA or GR-SU. Stock solutions (10 g/L) of chloramphenicol and tetracycline were prepared in ethanol. The final concentrations of gentamicin, chloramphenicol, tetracycline, oxacillin and ofloxacin were 32 mg/L, 32 mg/L, 64 mg/L, 128 mg/L, and 4 mg/L, respectively, and these solutions were then serially diluted 2-fold with the same media. After a 24 h incubation at 37°C, the MICs were determined as described above. The fractional inhibitory concentration (FIC) was calculated with a method previously described [34]. FIC indexes of ≤0.5, >0.5 – ≤4.0 and >4.0 were defined as synergy, no interaction and antagonism, respectively.

### Microarray analysis

Overnight cultures of *S*. *aureus* (10^8^ cells) were inoculated into 30 ml of fresh TSB and cultured at 37°C with shaking. When the OD_660_ reached 0.3, GR-SU (1x MIC: 128 mg/L) was added to the medium. After 2 h of incubation, the bacterial cells were collected by centrifugation at 5,000 x *g* for 5 min at 4°C and stored at -80°C until further use. Additionally, bacterial cells without GR-SU treatment were prepared as the control. Total RNA was extracted from the bacterial cells using the FastRNA Pro Blue Kit (MP Biomedicals, Cleveland, OH, USA) according to the manufacturer’s protocol. Then, cDNA was synthesized from 10 μg of total RNA using the FairPlay III Microarray Labeling Kit (Agilent Technologies, Santa Clara, CA, USA) according to the manufacturer’s instructions. The Agilent eArray platform (Agilent Technologies) was used to design the microarray; 13,939 probes (60-mers) were designed for the 2,628 protein-coding genes of *S*. *aureus* MW2 (up to five probes per gene). For microarray analyses, the test (GR-SU treatment) and control cDNAs were labeled with Cy3 mono-Reactive Dye and Cy5 mono-Reactive Dye (GE Healthcare, UK), respectively. The fluorescently labeled cDNAs were purified using the FairPlay III Microarray Labeling Kit (Agilent Technologies) The Cy3-labeled and Cy5-labeled cDNAs were mixed and hybridized onto an array using the Hi-RPM Gene Expression Hybridization Kit (Agilent Technologies). The arrays were scanned with an Agilent scanner, and data extraction, filtering and normalization were conducted using the Agilent Feature Extraction software according to the manufacturer’s instructions. The experiments were performed using two biological replicates (two technical replicates for each condition), and the expression data were deposited into the Gene Expression Omnibus (http://www.ncbi.nlm.nih.gov/geo/) under accession GSE80500. We compared difference between control and test sample for each gene by student t-test using Cyber-T (http://cybert.microarray.ics.uci.edu/). Factors that exhibited a greater than 2-fold change (increase/decrease) and *P<0*.*05* were defined as being altered by GR-SU.

### Gene expression analysis by quantitative PCR

Overnight cultures of *S*. *aureus* MW2 (10^8^ cells) were inoculated into 5 ml of TSB and aerobically incubated at 37°C with shaking. When the OD_660_ reached 0.3, either 1/4 or 1x MIC of GR-SU or GRA was added to the culture. The bacterial cells were collected after 1, 2, 4 and 8 h of incubation. RNA extraction and cDNA synthesis were performed as described above. Quantitative PCR was performed with the LightCycler Nano system (Roche, Tokyo, Japan) using cDNA as the template DNA. The primers used are shown in Table 1. The results were normalized to the housekeeping gene *gyrB*. Three independent experiments were performed, and the mean ± SE was calculated.

**Table 1:** Primers used in this study.

Gene name	Upper primer	Lower primer
For quantitative PCR		
*gyLB*	5'- AGGTCTTGGAGAAATGAATG 3'	5' -CAAATGTTTGGTCCGCTT 3'
*seh*	5' -TCAAGGTGATAGTGGCAAT 3'	5' -CCAATCACCCTTTCCTGT 3'
*clfB*	5' -TGCAAGTGCAGATTCCGA 3'	5' -TCATTTGTTGAAGCTGGCTC 3'
*sea*	5' -GATCAATTTATGGCTAGACG 3'	5' -CGAAGGTTCTGTAGAAGTATGA 3'
*sbi*	5' -CCAAGATAACAAAGCACCAC 3'	5' -TGCTGATTTCACACGCTC 3'
*sak*	5' -ACAGGCCCGTATTTGATG 3'	5' -GCCCATTCGACATAGTATTC 3'
*clfA*	5' -TACAAGTGCGCCTAGAATGA 3'	5' -TTTGACATAGCCTGCTTGGT 3'
*hlgC*	5' -ATTTCGTTCCAGACAGTGAG 3'	5' -CCGTCTAAATAACTGTTGCC 3'
*hla*	5' -GGGGACCATATGATAGAGATT 3'	5' -TGTAGCGAAGTCTGGTGAAA 3'
*coa*	5' -ACAGGGCACAATTACAGGT 3'	5' -GGTGTCGTCGGTTGAGTAT 3'
*lukS-PV*	5' -ATGAGGTGGCCTTTCCAATAC 3'	5' -CCTGTTGATGGACCACTATTA 3'
*nuc*	5' -AATCATACGGGTCCTTTCA 3'	5' -CCGTTTCTGGCGTATCAA 3'
*fnbA*	5' -TGAGGGTGGTTATGTTGATG 3'	5' -CAGTGTATCCTCCAACGTGA 3'
*icaA*	5' -AGTTGTCGACGTTGGCTAC 3'	5' -CCAAAGACCTCCCAATGT 3'

### Effect of GRA and GR-SU on carbohydrate and amino acids requirements

Chemically defined medium (CDM) and CDM containing 1/2 MIC or 1/4 MIC of GRA-K, GR-SU or GRA were used in this experiment. Overnight cultures of *S*. *aureus* MW2 were adjusted to 1x10^7^ cells/ml. Glucose, trehalose, sucrose or fructose (each 50 mM) was serially diluted 2-fold with either TSB or TSB containing GRA, K-GRA or GR-SU (1/2 MIC or 1/4 MIC at the final concentration for each agent). Then, 10 μl of the bacterial culture (1x10^5^ cells) was applied to each well. After incubation for 24 h at 37°C, the minimum concentration of each sugar that showed visible bacterial growth was determined.

To investigate the effect on amino acid requirement, we used CDM containing 18 amino acids necessary for *S*. *aureus* growth. The amino acids used in this experiment and their concentrations have been previously described [33]. The CDM was prepared using 2-fold serial dilutions with CDM depleted of all amino acids. Then, 10 μl of the bacterial culture (1x10^5^ cells) was applied to each well. After incubation for 24 h at 37°C, the minimum dilution rate that showed visible bacterial growth was determined.

## Results

### Antimicrobial activities of GRA and its derivatives against *S*. *aureus* strains

The MICs of GRA and its 4 derivatives are shown in Table 2. The MICs were evaluated using 50 *S*. *aureus* strains (19 MRSA strains and 31 MSSA strains). The MICs of GR-K and GR-S were higher than GA, GR-SU and GR-SA. The MICs of GR-K and GR-S against all strains were more than 256 mg/L. While GR-SA exhibited an intermediate level of MICs on the tested strains (ranged from 64 to 4,096 mg/L except 2 strains [more than 4,096 mg/L]), the MICs of GRA and GR-SU ranged from 16 to 512 mg/L and from 16 to 256 mg/L, respectively. The distribution of MICs in the MRSA and MSSA strains is shown in Table 2. We found no differences in susceptibility between the MRSA and MSSA strains.

**Table 2:** MIC of glycyrrhetinic acid and its derivatives against *S*.*aureus* strains.

	MIC (mg/L)				
strain	GR-K^a^	GR-SU^b^	GRA^c^	GR-S^d^	GR-SA^e^
MRSA					
SA5001	2048	128	64	>4096	1024
SA5002	256	16	64	>4096	512
SA5003	1024	32	32	>4096	1024
SA5004	>4096	64	64	>4096	1024
SA5007	2048	128	256	>4096	512
SA5008	4096	128	256	>4096	1024
SA5012	4096	256	512	>4096	2048
SA5013	4096	128	64	>4096	512
SA5021	2048	256	128	1024	512
SA5046	1024	64	512	512	512
SA5052	4096	128	512	512	512
SA5053	1024	16	32	2048	2048
SA5056	>4096	128	512	>4096	1024
SA5057	>4096	128	128	4096	512
SA5059	256	32	32	1024	1024
SA5060	4096	128	128	2048	2048
SA5065	4096	64	32	256	128
SA5070	4096	32	512	2048	512
MW2	2048	128	512	2048	1024
MSSA					
SA5006	>4096	32	64	>4096	512
SA5014	1024	32	128	1024	256
SA5015	2048	128	128	>4096	256
SA5016	1024	64	64	>4096	1024
SA5018	512	16	16	256	128
SA5019	1024	64	64	256	64
SA5020	2048	256	256	4096	4096
SA5022	2048	64	512	2048	512
SA5024	4096	256	512	4096	4096
SA5025	256	64	256	256	64
SA5026	1024	256	512	4096	2048
SA5027	512	256	512	4096	2048
SA5029	4096	256	512	2048	2048
SA5031	4096	64	128	>4096	>4096
SA5032	>4096	128	512	>4096	1024
SA5033	2048	256	512	2048	4096
SA5034	2048	128	512	256	512
SA5038	4096	256	512	4096	512
SA5039	2048	128	512	512	1024
SA5040	2048	64	256	512	512
SA5042	2048	32	256	1024	256
SA5043	1024	32	512	512	512
SA5044	4096	256	512	2048	2048
SA5062	4096	256	256	2048	2048
SA5063	4096	256	512	512	256
SA5064	4096	256	512	1024	1024
SA5066	2048	64	128	256	128
SA5067	1024	64	512	2048	512
SA5069	2048	128	256	>4096	1024
SA5071	2048	64	128	>4096	>4096
SA5073	2048	256	256	2048	1024

^a^Dipotassium glycyrrhizate

^b^disodium succinoyl glycyrrhetinate

^c^Glycyrrhetinic acid

^d^Stearyl glycyrrhetinate

^e^glycyrrhetinyl stearate

### Effects of GRA and GR-SU on *S*. *aureus* growth

**Fig 2:**
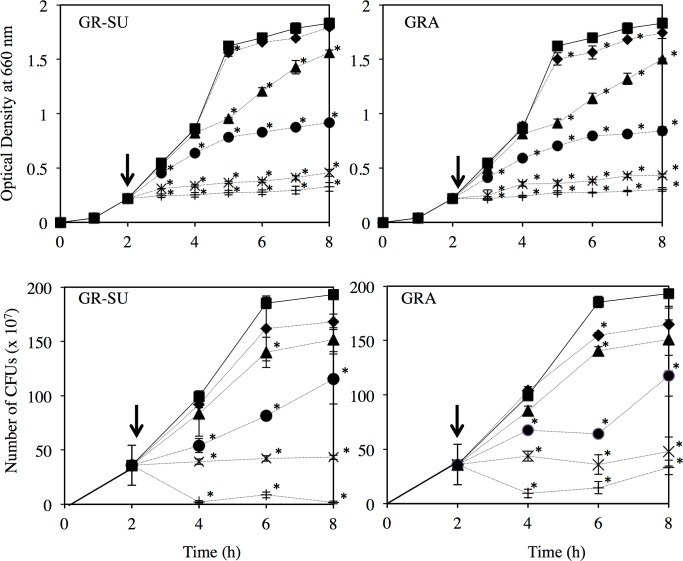
Effect of GRA and GRA derivatives on *S*. *aureus* MW2 growth. Overnight cultures of *S*. *aureus* MW2 were adjusted to an OD of 1.0. Then, 100 μl of bacterial culture was inoculated into 5 ml of TSB. The bacterial culture was incubated at 37°C with shaking. When the OD at 660 nm reached 0.3, various concentrations (■: control, ♦: 1/64 MIC, ▲: 1/16 MIC, ●: 1/4 MIC, ×: 1x MIC, +: 2x MIC) of GR-SU or GRA were added to the medium. The growth and colony counts were monitored. Three independent experiments were performed, and the mean ± SD was calculated. The data were analyzed for statistically significant differences compared to untreated control by a two-way ANOVA followed by Dunnett’s post hoc tests. *P<0.05.

As shown in Fig 2, *S*. *aureus* MW2 growth was inhibited by the addition of either GRA or GR-SU in a dose-dependent manner. The addition of more than 1x MIC of GRA or GR-SU fully inhibited the growth of *S*. *aureus* MW2 as expected, but growth was already inhibited by both agents at sub-MIC concentrations (1/16 and 1/4 MIC). We also investigated the effects of GRA and GR-SU on cell viability in a time course experiment (Fig 2). The result was similar to that obtained with the OD. The viable cell number was suppressed by the addition of either GRA or GR-SU in a dose-dependent manner. The bacterial cell numbers were decreased after 4 h by the addition of 2x MIC of GRA or GR-SU. Additionally, we analyzed a methicillin-sensitive strain (MS23513) and obtained similar findings (S1 Fig).

### Effects of GRA and GR-SU on cell viability

**Fig 3:**
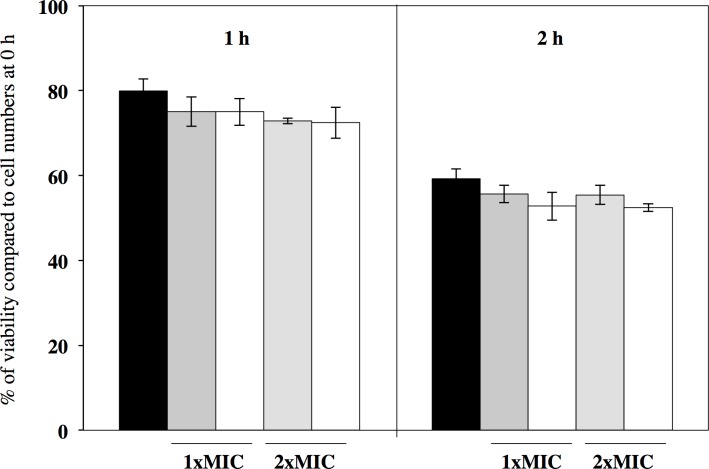
Effect of GRA and GRA derivatives on *S*. *aureus* cell viability under non-growing conditions. Cell viability was evaluated under non-growing conditions following the addition of GRA or GR-SU. The methods are described in the Materials and Methods section. *S*. *aureus* MW2 was treated with 1x MIC and 2x MIC of GR-SU (grey bar) or GRA (white bar) in 10 mM sodium phosphate buffer. An untreated bacterial culture (black bar) was used as a control. After 1 and 2 h of incubation, the reaction mixtures (100 μl) were plated on trypticase soy agar and incubated at 37°C overnight. The colony forming units (CFUs) were determined as the total number of colonies identified on each plate. The antibacterial effect was calculated as the ratio of the number of surviving cells (survival rate %) to the total number of bacteria incubated. Three independent experiments were performed, and the mean ± SD was calculated. The data were analyzed for statistically significant differences compared to the untreated control by a two-way ANOVA followed by Dunnett’s post hoc test. Significant differences were not observed.

Because GRA and GR-SU exhibited antimicrobial activity against *S*. *aureus* strains during active growth, we investigated whether this activity was observed under non-growing conditions. We treated *S*. *aureus* MW2 cells with either GRA or GR-SU in 10 mM PB. As shown in Fig 3, the viable cell number of the MW2 strain treated with 1x MIC and 2x MIC of GRA or GR-SU showed no significant difference compared to the control (no treatment).

### Synergistic effects of GRA and GR-SU against 8 *S*. *aureus* strains

Next, we evaluated the synergistic effects of GRA and GR-SU in combination with other antibiotics. As shown in Table 3, all strains tested showed synergy with the combination of gentamicin with either GRA or GR-SU, while one strain showed synergy with the combination of tetracycline or chloramphenicol with GR-SU, and 2 strains showed synergy with the combination of oxacillin with GRA. Oxacillin, ofloxacin and erythromycin had no effect (no interaction) when combined with GR-SU, and chloramphenicol, ofloxacin and erythromycin had no effect when combined with GRA. Furthermore, the MICs of antibiotics in the absence or presence of GRA or GR-SU are shown in S2 Table. A synergistic effect of GRA and GR-SU with gentamycin against both gentamicin-resistant (4 to 128 mg/L) and susceptible strains (3 strains with MIC of 1 mg/L) was found (S2 Table). In the presence of 1/2 MIC of GRA, 4 strains exhibited a 2- to 8-fold decrease in the MIC of oxacillin, while 4 other strains exhibited no change. In the presence of the 1/4 MIC of GRA, 2 strains also exhibited an 8-fold decrease in the MIC of oxacillin. In the presence of 1/2 and 1/4 MICs of GRA or GR-SU, all strains exhibited a 4- to 8-fold decrease in the MIC of gentamicin. In the presence of the 1/8 MICs of GRA or GR-SU, increased MICs were not observed in any of the antibiotics tested in this study (S2 Table).

**Table 3:** FICIs in combination of various antibiotics with GRA or GR-SU.

	Number of strains					
	FICI^a^ (GR-SU)			FICI (GRA)		
Antibiotics	≤0.5	>0.5~≤4.0	>4.0	≤0.5	>0.5~≤4.0	>4.0
Oxacillin	0	8	0	2	6	0
tetracycline	1	7	0	0	8	0
gentamicin	8	0	0	8	0	0
chloramphenicol	1	7	0	0	8	0
ofloxacin	0	8	0	0	8	0
erythromycin	0	8	0	0	8	0

FICI: ≤0.5; synergy, >0.5~≤4.0; no interaction, >4.0; antagonism

### Microarray analysis

**Fig 4:**
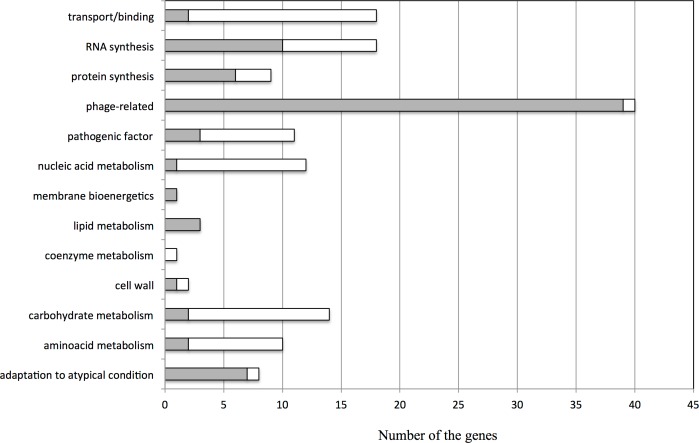
Microarray analysis of genes with altered expression levels in response to GR-SU. The microarray analysis was performed as described in the Materials and Methods section. The numbers of genes with 2-fold upregulated (grey bar) or downregulated (white bar) expression are indicated.

We performed a microarray analysis to identify genes with altered expression patterns in response to GR-SU. As summarized in Fig 4, more than 200 genes exhibited altered expression levels in response to GR-SU. Among these, 39 phage-related genes were increased by the addition of GR-SU. In the *S*. *aureus* MW2 genome, two phage-related operons (MW1380-MW1451 and MW1882-MW1952) were found. Specifically, in the operon of MW1380-MW1451 (72 genes), the expression of 31 genes was increased. Many genes responsible for metabolic and biosynthetic pathways also exhibited altered expression patterns (S3–S6 Tables). The expression levels of 6 genes involved in protein synthesis, 10 genes in RNA synthesis and 2 genes in amino acid metabolism were increased, whereas the levels of 8 genes for RNA synthesis, 12 genes for carbohydrate metabolism and 8 genes for amino acid metabolism were decreased. In addition, the expression levels of 2 transporter and substrate-binding protein genes were increased, and 16 genes were decreased after GR-SU treatment. Regarding pathogenic factors, the expression of 3 factors was increased while that of 8 factors was decreased.

### Effect of GRA and GR-SU on virulence factor expression

**Fig 5:**
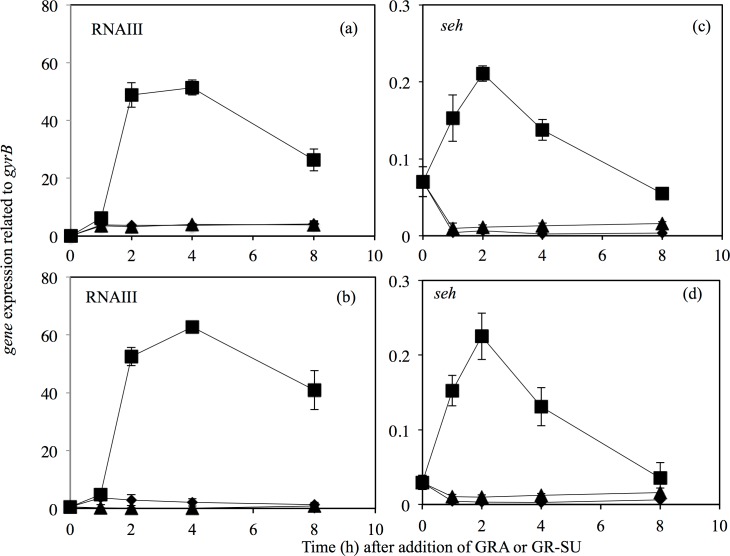
**Effects of GRA and GRA derivatives on the expression of RNAIII (a, b) and *seh* (c, d).** Overnight cultures of *S*. *aureus* MW2 (10^8^ cells) were inoculated into 5 ml of TSB and aerobically incubated at 37°C with shaking. When the OD at 660 nm reached 0.3, 1/4 MIC (a, c) or 1x MIC (b, d) of GR-SU or GRA was added to the culture (■: control, ♦: GR-SU, ▲: GRA). Bacterial cells were collected after 1, 2, 4 and 8 h of incubation. Then, RNA extraction and cDNA synthesis were performed. Quantitative PCR was performed using cDNA as the template DNA. Three independent experiments were performed and the mean ± SE was calculated.

As we found in the microarray analysis, the expression levels of several virulence-related genes were altered in response to GR-SU. We analyzed the gene expression of RNAIII, which is a key factor involved in the expression of many virulence genes, in more detail by quantitative PCR. As shown in Fig 5A and 5B, RNAIII expression was significantly increased during the exponential phase in the absence of GRA or GR-SU, whereas GRA and GR-SU both fully suppressed RNAIII expression at 1/4 MIC. The expression of *seh*, which is a RNAIII-dependent virulence factor, exhibited a pattern similar to that of RNAIII in response to GRA and GR-SU (Fig 5C and 5D). These results suggest that the repression of RNAIII by GR-SU may explain the alterations in gene expression of several virulence-related genes, which were observed in the microarray analysis (Fig 4).

**Fig 6:**
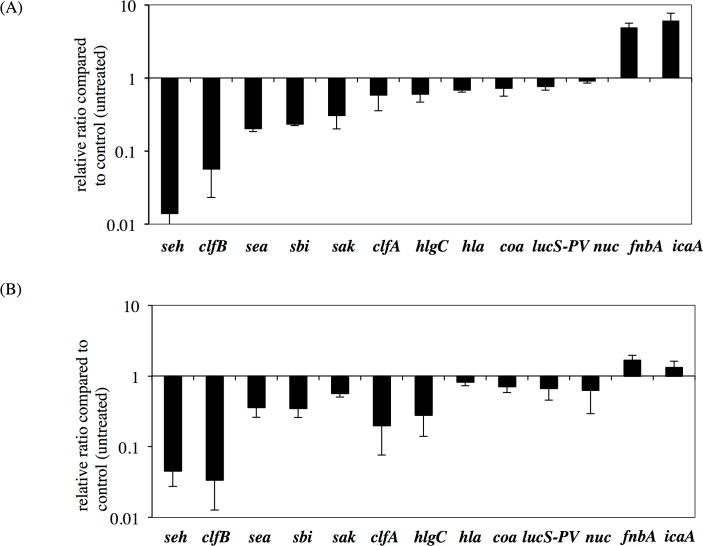
Effects of GRA and GRA derivatives on virulence factor expression. Overnight cultures of *S*. *aureus* MW2 (10^8^ cells) were inoculated into 5 ml of TSB and aerobically incubated at 37°C with shaking. When the OD at 660 nm reached 0.3, 1x MIC of GR-SU (a) or GRA (b) was added to the culture. The bacterial cells were collected after 2 h. Then, RNA extraction and cDNA synthesis were performed. A quantitative PCR was performed using cDNA as the template DNA. Three independent experiments were performed and the mean ± SE was calculated.

Therefore, we investigated the expression levels of 12 virulence factors and found that several toxins such as *sea*, *clfAB* and *hlgC* were downregulated by GRA, whereas the cell surface factors *fnbA* and *ica* were upregulated (Fig 6). The effect of GR-SU on virulence factor expression was similar to that of GRA.

### Effects of GRA and GR-SU on sugar and amino acid requirements

Because amino acid and carbohydrate metabolism tended to decrease following a 2-hour exposure to GRA and GR-SU based on the microarray analysis, we hypothesized that GRA and GR-SU may inhibit the growth of *S*. *aureus* by influencing amino acid and carbohydrate metabolism. Therefore, we investigated sugar and amino acid requirements in the presence of GRA, GR-SU or GRA-K (1/2 MIC or 1/4 MIC at the final concentration for each agent). As shown in Table 4, the minimum concentrations of each sugar for the growth of *S*. *aureus* MW2 in CDM containing glucose, sucrose, lactose or trehalose was increased by 2- or 4-fold in the presence of 1/2 MIC of GRA or GR-SU, whereas GRA-K did not increase the sugar requirement. Additionally, we investigated the amino acid requirements. GRA and GR-SU treatment caused a 2-fold higher requirement compared to untreated bacteria. When 1/4 MIC of GRA or GR-SU was used, the minimal concentration of each sugar or amino acids was not altered.

**Table 4:** Effect of GRA and GR-SU on sugar and amino acids requirement.

Minimum concentration for growth (mM: except for amino acids)					
GRA derivatives	glucose	sucrose	lactose	trehalose	Amino acids^a^
none	0.78	0.39	0.39	0.39	1/32^b^
GR-K	0.78	0.39	0.39	0.39	1/32
GR-SU	3.13	1.56	1.56	1.56	1/16
GRA	3.13	0.78	0.78	0.78	1/16

a: dilution ratio from CDM (1x amino acids)

## Discussion

In this study, we demonstrated the antibacterial activities of GRA and GR-SU against *S*. *aureus* strains. These two compounds exhibited strong antibacterial activities, but the antibacterial activities of the other GRA derivatives (GR-S, GR-K and GR-SA) were much weaker than those of GRA and GR-SU. GR-SU is synthesized from GRA by adding a succinic acid moiety to increase water solubility. GR-K (dipotassium glycyrrhizate) is a potassium salt of glycyrrhizin (GRA with two glucuronic acids) and also has high water solubility. Both GR-SU and GR-K have high water solubility, but the antibacterial effect of GR-K is much lower than that of GR-SU. Thus, difference in water solubility is not related to the difference in antibacterial activity among these compounds. Instead, some other structural differences between the two agents (Fig 1) may affect their antibacterial activities against *S*. *aureus* strains. GRA and GR-SU have been clinically used as anti-inflammatory agents [21, 32]. Taken together with our findings, GRA and GR-SU are expected to exert two biological activities of clinical importance: antibacterial and anti-inflammatory activities.

Although several investigations regarding the antibacterial effects of GRA have been reported [28, 29], this is the first report of the use of GR-SU as an antibacterial agent. Glycyrrhetinic acid derivatives are known anti-inflammatory drugs [30, 31, 32]. Stronger Neo-Minophagen C, which is the most well-known glycyrrhetinic acid derivative drug, contains 2 mg/ml of monoammonium glycyrrhizinate hydrate at the dose used to clinically treat chronic hepatitis; this dose is approximately 4- to 20-fold higher than the concentration used in this study [26, 27]. Therefore, GRA and GR-SU may have a potential use against *S*. *aureus* infectious diseases caused by antibacterial and anti-inflammatory effects.

Long, D.R. *et al*. previously reported the bacteriostatic activity of GRA and the bactericidal effect of GRA at a high concentration (above 62.5 mg/L) against *S*. *aureus* [28]. In this study, we also observed bactericidal activity of GRA and GR-SU at a 2x MIC dose (GR-SU: 256 mg/L, GRA: 1,024 mg/L). As shown in the microarray analysis (Fig 4), the expression levels of many phage-related genes were increased by the addition of 1x MIC of GR-SU, and we speculated that the bactericidal effect of a high dose of GR-SU (2xMIC) was due to phage induction-mediated cell lysis.

Among the genes that exhibited altered expression following the addition of GR-SU, many genes involved in sugar (S3 Table), amino acid (S4 Table) and nucleic acid (S5 Table) metabolism were significantly decreased. Moreover, the expression levels of many genes that encoded transporters were also decreased (S6 Table). These results suggest that GRA and GR-SU-treatments inhibit the uptake of nutrients, which then causes alterations in the expression of genes related to nutrient metabolism. Supporting this hypothesis, *S*. *aureus* MW2 required a higher concentration of sugars and amino acids in the presence of GRA and GR-SU than in the absence of these compounds (Table 4). Based on these findings, we propose that GRA and GR-SU have no specific mechanism for antibacterial activity but induce antimicrobial effects by inhibiting nutrient acquisition and affecting bacterial metabolism. Additionally, the expression of several transporters responsible for the efflux and/or influx of compounds was increased in the microarray analysis. The transporters with altered expression levels in the presence of either GRA or GR-SU included some transporters that might be involved in the high susceptibility to gentamicin in the presence of either GRA or GR-SU. GRA and GR-SU had weak synergistic activities with other protein synthesis inhibitors, speculating that transporters specific for gentamicin influx may be influenced by GRA and GR-SU.

In this study, we found that the expression of RNAIII, which is responsible for the regulation of many *S*. *aureus* virulence factors, was fully repressed by GRA and GR-SU [35, 36]. Because RNAIII is related to the *agr* quorum sensing system, RNAIII expression is growth dependent [35]. We found that GRA and GR-SU suppressed growth in a dose-dependent manner, suggesting that RNAIII expression was inhibited. Then, we investigated the expression of several virulence factors in the presence or absence of GRA or GR-SU. As shown in Fig 6, the expression levels of toxins such as enterotoxins (*seh* and *sea*), staphylokinase (*sak*) and *hlgC* were decreased, whereas the expression levels of cell surface factors such as *fnbA* and *ica* were increased. From these results, we hypothesize that GRA and GR-SU can modulate virulence in addition to their antimicrobial effects on *S*. *aureus*.

In conclusion, we have found indications that the antibacterial activity of GRA and GR-SU are mostly occurring via effects on nutrient metabolism. Additionally, these agents suppressed the expression of several virulence genes. Taken together with previous reports of inflammatory effects, GRA and GR-SU may have potential for clinical use against *S*. *aureus* infectious diseases.

## Supporting Information

S1 Fig Effect of GRA and GRA derivatives on *S*. *aureus* growth. Overnight cultures of *S*. *aureus* MS23513 were adjusted to an OD at 660 nm of 1.0. Then, 100 μl of bacterial culture was inoculated in 5 ml TSB. Bacterial cultures were incubated at 37°C with shaking. When the OD at 660 nm reached 0.3, various concentrations (■: control; ♦: 1/64 MIC; ▲:1/16 MIC; ●: 1/4 MIC; ×: 1x MIC; 十: 2x MIC) of GR-SU or GRA were added to the medium. Growth and colony counts were monitored during growth. Three independent experiments were performed, and the mean ± SD was calculated. The data were analyzed for statistically significant differences compared to untreated control for each condition by a two-way ANOVA followed by Dunnett’s post hoc tests. *P<0.05.
(PPTX)

S1 Table *S*.*aureus* clinical strains. (DOCX)

S2 Table Effect of sub-MIC GRA and GR-SU on the susceptibility to various antibiotics. (DOCX)

S3 Table Genes for carbohydrate metabolism up- and down-regulated by GR-SU. (DOCX)

S4 Table Genes for amino acids metabolism up- and down-regulated by GR-SU. (DOCX)

S5 Table Genes for nucleic acids metabolism up- and down-regulated by GR-SU. (DOCX)

S6 Table Genes for transporters up- and down-regulated by GR-SU. (DOCX)
