# Supplementary material for: Antibacterial Effects of Glycyrrhetinic Acid and Its Derivatives on Staphylococcus aureus
Source: PLoS One. 2016 Nov 7;11(11):e0165831. doi: 10.1371/journal.pone.0165831 (PMC5098735; doi:10.1371/journal.pone.0165831)
Supplement: S1 Fig — Overnight cultures of S. aureus MS23513 were adjusted to an OD at 660 nm of 1.0. Then, 100 μl of bacterial culture was inoculated in 5 ml TSB. Bacterial cultures were incubated at 37°C with shaking. When the OD at 660 nm reached 0.3, various concentrations (■: control; ♦: 1/64 MIC; ▲:1/16 MIC; ●: 1/4 MIC; ×: 1x MIC; 十: 2x MIC) of GR-SU or GRA were added to the medium. Growth and colony counts were monitored during growth. Three independent experiments were performed, and the mean ± SD was calculated. The data were analyzed for statistically significant differences compared to untreated control for each condition by a two-way ANOVA followed by Dunnett’s post hoc tests. *P<0.05. (PPTX) [file pone.0165831.s001.pptx]

## Slide 1
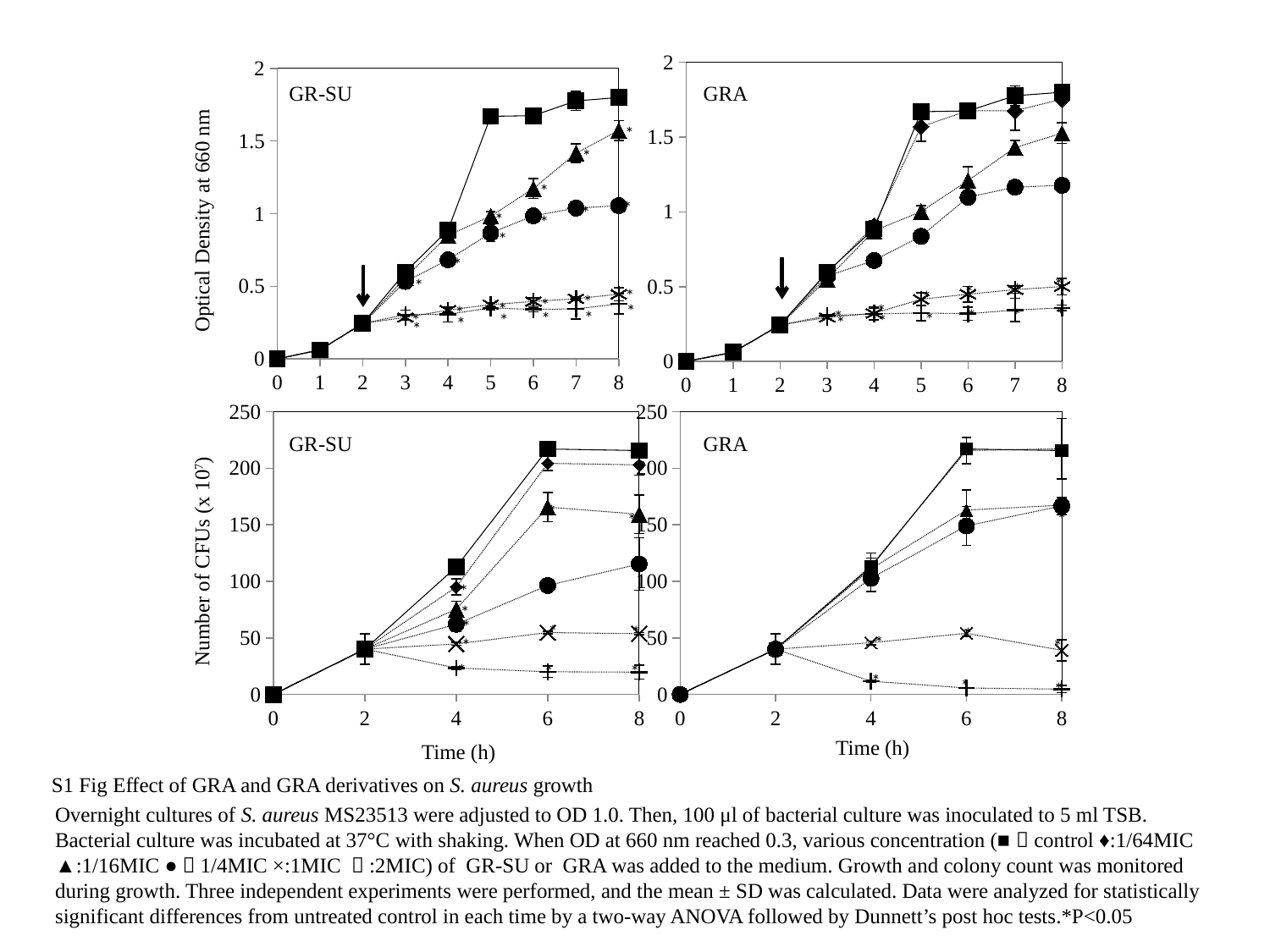

### Chart
| Category | con | 1/64MIC | 1/16MIC | 1/4MIC | 1MIC | 2MIC |
|---|---|---|---|---|---|---|
| 0.0 | 0.0 | 0.0 | 0.0 | 0.0 | 0.0 | 0.0 |
| 1.0 | 0.0613333333333333 | 0.0613333333333333 | 0.0613333333333333 | 0.0613333333333333 | 0.0613333333333333 | 0.0613333333333333 |
| 2.0 | 0.243333333333333 | 0.243333333333333 | 0.243333333333333 | 0.243333333333333 | 0.243333333333333 | 0.243333333333333 |
| 3.0 | 0.597666666666667 | 0.590666666666667 | 0.550666666666667 | 0.570666666666667 | 0.294666666666667 | 0.305666666666667 |
| 4.0 | 0.886666666666667 | 0.908 | 0.873333333333333 | 0.675 | 0.321666666666667 | 0.318333333333333 |
| 5.0 | 1.67 | 1.57 | 1.002 | 0.837 | 0.415666666666667 | 0.324 |
| 6.0 | 1.675 | 1.676666666666666 | 1.21 | 1.098333333333333 | 0.449666666666667 | 0.319 |
| 7.0 | 1.776666666666667 | 1.676666666666667 | 1.43 | 1.165666666666667 | 0.480333333333333 | 0.343666666666667 |
| 8.0 | 1.8 | 1.753333333333333 | 1.526666666666667 | 1.178 | 0.500666666666667 | 0.358 |
### Chart
| Category | con | 1/16MIC | 1/4MIC | 1MIC | 2MIC |
|---|---|---|---|---|---|
| 0.0 | 0.0 | 0.0 | 0.0 | 0.0 | 0.0 |
| 1.0 | 0.0613333333333333 | 0.0613333333333333 | 0.0613333333333333 | 0.0613333333333333 | 0.0613333333333333 |
| 2.0 | 0.243333333333333 | 0.243333333333333 | 0.243333333333333 | 0.243333333333333 | 0.243333333333333 |
| 3.0 | 0.597666666666667 | 0.559333333333333 | 0.532666666666667 | 0.285333333333333 | 0.304 |
| 4.0 | 0.886666666666667 | 0.849333333333333 | 0.681666666666667 | 0.335333333333333 | 0.306666666666667 |
| 5.0 | 1.67 | 0.983 | 0.866666666666667 | 0.373666666666667 | 0.350333333333333 |
| 6.0 | 1.675 | 1.173 | 0.985 | 0.395 | 0.34 |
| 7.0 | 1.776666666666667 | 1.416666666666667 | 1.038333333333333 | 0.414333333333333 | 0.344 |
| 8.0 | 1.8 | 1.573333333333333 | 1.055 | 0.444666666666667 | 0.378666666666667 |GR-SU
GRA
*
*
*
*
*
*
*
*
*
*
*
*
*
*
Optical Density at 660 nm
*
*
*
*
*
*
*
*
*
*
*
*
*
*
*
*
*
*
*
*
*
*
*
*
*
*
*
*
*
### Chart
| Category | (-) | ② 2MIC | ② 1MIC | ② 1/4MIC | ② 1/16MIC | ② 1/64MIC |
|---|---|---|---|---|---|---|
| 0.0 | 0.0 | 0.0 | 0.0 | 0.0 | 0.0 | 0.0 |
| 2.0 | 40.0 | 40.0 | 40.0 | 40.0 | 40.0 | 40.0 |
| 4.0 | 112.6666666666667 | 23.33333333333328 | 44.66666666666641 | 62.0 | 75.3333333333331 | 95.0 |
| 6.0 | 217.0 | 20.0 | 54.66666666666641 | 96.3333333333331 | 165.6666666666667 | 204.3333333333333 |
| 8.0 | 215.6666666666667 | 19.66666666666667 | 53.66666666666641 | 115.3333333333333 | 159.3333333333333 | 203.0 |
### Chart
| Category | (-) | ③ 2MIC | ③ 1MIC | ③1/4MIC | ③ 1/16MIC | ③ 1/64MIC |
|---|---|---|---|---|---|---|
| 0.0 | 0.0 | 0.0 | 0.0 | 0.0 | 0.0 | 0.0 |
| 2.0 | 40.0 | 40.0 | 40.0 | 40.0 | 40.0 | 40.0 |
| 4.0 | 112.6666666666667 | 11.66666666666667 | 45.66666666666641 | 102.6666666666667 | 111.0 | 113.6666666666667 |
| 6.0 | 217.0 | 5.666666666666667 | 54.0 | 149.0 | 163.0 | 215.6666666666667 |
| 8.0 | 215.6666666666667 | 4.666666666666667 | 39.0 | 166.6666666666667 | 167.3333333333333 | 217.3333333333333 |GR-SU
GRA
*
*
*
*
*
*
Number of CFUs (x 107)
*
*
*
*
*
*
*
*
*
*
*
*
*
*
*
*
*
Time (h)
Time (h)
 S1 Fig Effect of GRA and GRA derivatives on S. aureus growth
Overnight cultures of S. aureus MS23513 were adjusted to OD 1.0. Then, 100 μl of bacterial culture was inoculated to 5 ml TSB. Bacterial culture was incubated at 37°C with shaking. When OD at 660 nm reached 0.3, various concentration (■：control ♦︎:1/64MIC ▲:1/16MIC ●：1/4MIC ×:1MIC 十:2MIC) of GR-SU or GRA was added to the medium. Growth and colony count was monitored during growth. Three independent experiments were performed, and the mean ± SD was calculated. Data were analyzed for statistically significant differences from untreated control in each time by a two-way ANOVA followed by Dunnett’s post hoc tests.*P<0.05
