# Supplementary material for: Antibacterial Effects of Glycyrrhetinic Acid and Its Derivatives on Staphylococcus aureus
Source: PLoS One. 2016 Nov 7;11(11):e0165831. doi: 10.1371/journal.pone.0165831 (PMC5098735; doi:10.1371/journal.pone.0165831)
Supplement: S1 Table — (DOCX) [file pone.0165831.s002.docx]

S1 Table *S.aureus* clinical strains

| Strains | Character |
| --- | --- |
| MRSA | |
| MW2 | sepsis |
| SA5001(MS23183) | sepsis |
| SA5002(MS23143) | sepsis |
| SA5003(MS23105) | sepsis |
| SA5004(MS23304) | sepsis |
| SA5007(MS23543) | sepsis |
| SA5008(TF2988) | pancreatitis |
| SA5012(633 TOKAI) | sepsis |
| SA5013(832 TOKAI) | sepsis |
| SA5059(TY254) | Subglottic stenosis |
| SA5021(TY343) | NTED |
| SA5046(TY186) | SSSS |
| SA5057(TF3303) | sepsis |
| SA5060(TF3345) | Subglottic stenosis |
| SA5052(TF2826) | skin |
| SA5053(TF338) | unknown |
| SA5065(TY34) | imperigo |
| SA5056(MS23002) | sepsis |
| SA5070(TF3030) | in supprative infection |
| MSSA | |
| SA5006(MS23513) | sepsis |
| SA5014(TF2756) | sepsis |
| SA5015(TF2890) | atopy |
| SA5016(TF3001) | atopy |
| SA5018(TF3340) | SSSS |
| SA5019(TY507) | SSSS |
| SA5020(TY526) | impetigo |
| SA5022(TY234) | SSSS |
| SA5024(TY1159) | impetigo |
| SA5025(TY1638) | impetigo |
| SA5026(TY1741) | impetigo |
| SA5027(TY1800) | impetigo |
| SA5029(TF3053) | atopy |
| SA5031(TY1355) | SSSS |
| SA5033(TF2870) | atopy |
| SA5034(TF2856) | atopy |
| SA5044(TF2867) | atopy |
| SA5038(TF2805) | atopy |
| SA5042(TF3343) | unknown |
| SA5063(TY1073) | sepsis |
| SA5064(TY780) | impetigo |
| SA5062(MS23488) | sepsis |
| SA5073(TF3483) | sepsis |
| SA5066(TY964) | impetigo |
| SA5071(TF3453) | sepsis |
| SA5067(TY575) | impetigo |
| SA5043(TY176) | atopy |
| SA5040(TF2810) | atopy |
| SA5069(TY730) | impetigo |
| SA5032(TF2851) | atopy |
| SA5039(TY958) | impetigo |
