# Supplementary material for: Antibacterial Effects of Glycyrrhetinic Acid and Its Derivatives on Staphylococcus aureus
Source: PLoS One. 2016 Nov 7;11(11):e0165831. doi: 10.1371/journal.pone.0165831 (PMC5098735; doi:10.1371/journal.pone.0165831)
Supplement: S2 Table — (DOCX) [file pone.0165831.s003.docx]

S2 Table. Effect of sub-MIC GRA and GR-SU on the susceptibility to various antibiotics

(a)MIC of antibiotics to 8 *S.aureus* strains

|  | MIC (mg/L) | | | | | |
| --- | --- | --- | --- | --- | --- | --- |
| Strain | MPIPC | TC | GM | CP | OFLX | EM |
| MW2 | 8 | 0.25 | 4 | 4 | 0.5 | 1 |
| SA5001 | 512 | 64 | 1 | 8 | 16 | 64 |
| SA5004 | 512 | 0.25 | 16 | 4 | 32 | 64 |
| SA5006 | 1 | 0.25 | 1 | 8 | 0.5 | 0.25 |
| SA5008 | 512 | 0.25 | 128 | 8 | 32 | 64 |
| SA5012 | 512 | 32 | 128 | 16 | 32 | 64 |
| SA5013 | 512 | 32 | 1 | 8 | 32 | 64 |
| SA5015 | 1 | 16 | 32 | 8 | 32 | 64 |

(b) MIC of antibiotics in the presence of sub-MIC GR-SU and GRA

|  | MIC (mg/L) | | | | | | | | | | | | |
| --- | --- | --- | --- | --- | --- | --- | --- | --- | --- | --- | --- | --- | --- |
| Strain | 1/8MIC GR-SU | | | | | |  | 1/8MIC GRA | | | | | |
|  | MPIPC | TC | GM | CP | OFLX | EM |  | MPIPC | TC | GM | CP | OFLX | EM |
| MW2 | 8 | 0.25 | 4 | 4 | 0.5 | 1 |  | 8 | 0.25 | 4 | 4 | 0.5 | 1 |
| SA5001 | 512 | 64 | 1 | 8 | 16 | 64 |  | 512 | 64 | 1 | 8 | 16 | 64 |
| SA5004 | 512 | 0.25 | 16 | 4 | 32 | 64 |  | 512 | 0.25 | 16 | 4 | 32 | 64 |
| SA5006 | 1 | 0.25 | 1 | 8 | 0.5 | 0.25 |  | 1 | 0.25 | 1 | 8 | 0.5 | 0.25 |
| SA5008 | 512 | 0.25 | 128 | 8 | 32 | 64 |  | 512 | 0.25 | 128 | 8 | 32 | 64 |
| SA5012 | 512 | 32 | 128 | 16 | 32 | 64 |  | 512 | 32 | 128 | 16 | 32 | 64 |
| SA5013 | 512 | 32 | 1 | 8 | 32 | 64 |  | 512 | 32 | 1 | 8 | 32 | 64 |
| SA5015 | 1 | 16 | 32 | 8 | 32 | 64 |  | 1 | 16 | 32 | 8 | 32 | 64 |

|  | MIC (mg/L) | | | | | | | | | | | | |
| --- | --- | --- | --- | --- | --- | --- | --- | --- | --- | --- | --- | --- | --- |
| Strain | 1/4MIC GR-SU | | | | | |  | 1/4MIC GRA | | | | | |
|  | MPIPC | TC | GM | CP | OFLX | EM |  | MPIPC | TC | GM | CP | OFLX | EM |
| MW2 | 8 | 0.25 | 1^a^ | 4 | 0.5 | 1 |  | 8 | 0.25 | 0.125^a^ | 4 | 0.5 | 1 |
| SA5001 | 512 | 16^a^ | 0.25^a^ | 4 | 16 | 64 |  | 64^a^ | 32 | 0.125^a^ | 8 | 16 | 64 |
| SA5004 | 512 | 0.25 | 4^a^ | 4 | 32 | 64 |  | 512 | 0.125 | 0.125^a^ | 4 | 32 | 64 |
| SA5006 | 1 | 0.25 | 0.25^a^ | 4 | 0.5 | 0.25 |  | 1 | 0.25 | 0.125^a^ | 8 | 0.5 | 0.25 |
| SA5008 | 512 | 0.125 | 16^a^ | 4 | 32 | 64 |  | 64^a^ | 0.125 | 1^a^ | 8 | 32 | 64 |
| SA5012 | 512 | 16 | 32^a^ | 8 | 32 | 64 |  | 512 | 16 | 1^a^ | 16 | 32 | 64 |
| SA5013 | 512 | 32 | 0.25^a^ | 4 | 32 | 64 |  | 512 | 16 | 0.125^a^ | 8 | 32 | 64 |
| SA5015 | 1 | 16 | 8^a^ | 2^a^ | 32 | 64 |  | 1 | 16 | 8^a^ | 8 | 32 | 64 |

a:synergistic effect

|  | MIC (mg/L) | | | | | | | | | | | | |
| --- | --- | --- | --- | --- | --- | --- | --- | --- | --- | --- | --- | --- | --- |
| Strain | 1/2MIC GR-SU | | | | | |  | 1/2MIC GRA | | | | | |
|  | MPIPC | TC | GM | CP | OFLX | EM |  | MPIPC | TC | GM | CP | OFLX | EM |
| MW2 | 8 | 0.125 | 0.5 | 2 | 0.5 | 1 |  | 8 | 0.25 | 0.125 | 4 | 0.5 | 1 |
| SA5001 | 512 | 16 | 0.25 | 2 | 16 | 64 |  | 64 | 32 | 0.125 | 8 | 16 | 64 |
| SA5004 | 512 | 0.25 | 4 | 2 | 32 | 64 |  | 64 | 0.125 | 0.125 | 4 | 32 | 64 |
| SA5006 | 1 | 0.25 | 0.125 | 4 | 0.5 | 0.25 |  | 1 | 0.125 | 0.125 | 8 | 0.5 | 0.25 |
| SA5008 | 512 | 0.125 | 32 | 4 | 32 | 64 |  | 64 | 0.125 | 1 | 8 | 32 | 64 |
| SA5012 | 512 | 16 | 4 | 8 | 32 | 64 |  | 512 | 16 | 1 | 16 | 32 | 64 |
| SA5013 | 512 | 32 | 0.25 | 4 | 32 | 64 |  | 256 | 16 | 0.125 | 8 | 32 | 64 |
| SA5015 | 1 | 16 | 8 | 2 | 32 | 64 |  | 1 | 16 | 8 | 8 | 32 | 64 |
