# Supplementary material for: Antibacterial Effects of Glycyrrhetinic Acid and Its Derivatives on Staphylococcus aureus
Source: PLoS One. 2016 Nov 7;11(11):e0165831. doi: 10.1371/journal.pone.0165831 (PMC5098735; doi:10.1371/journal.pone.0165831)
Supplement: S3 Table — (DOCX) [file pone.0165831.s004.docx]

S3 Table. Genes for carbohydrate metabolism up- and down-regulated by GR-SU

| Gene ID^a^ | Fold change^b^ | *P* value^c^ | characteristics |
| --- | --- | --- | --- |
| Down | | | |
| MW2132 | 0.24 | 0.041 | alpha-acetolactate synthase (*alsS*) |
| MW0217 | 0.27 | 0.004 | L-lactate dehydrogenase |
| MW0997 | 0.30 | 0.008 | pyruvate carboxylase (*pycA*) |
| MW2521 | 0.33 | 0.005 | L-lactate dehydrogenase (*ldh*) |
| MW1641 | 0.34 | 0.002 | hypothetical protein, similar to alpha-acetolactate decarboxylase |
| MW0812 | 0.40 | 0.002 | hypothetical protein, similar to glycerate dehydrogenase |
| MW0650 | 0.41 | 0.008 | hypothetical protein, similar to aryl-alcohol dehydrogenase |
| MW1642 | 0.42 | 0.038 | 6-phosphofructokinase (*pfk*) |
| MW0811 | 0.47 | 0.008 | hypothetical protein, similar to aryl-alcohol dehydrogenase |
| MW2049 | 0.47 | 0.013 | fructose-bisphosphate aldolase (*fbaA*) |
| MW1654 | 0.48 | 0.001 | acetate kinase homolog (*ackA*) |
| MW0228 | 0.49 | 0.015 | hypothetical protein, similar to xylitol dehydrogenase |
| Up | | | |
| MW2366 | 2.49 | 0.008 | hypothetical protein, similar to pyruvate oxidase |
| MW2379 | 2.21 | 0.028 | hypothetical protein, similar to endo-1,4-beta-glucanase |

^a^Based on the sequence of MW2 strain (accession no: NC_003923.1).

^b^”UP” represents GR-SU decreased the expression at more than 2 fold compared with that without treatment, while “Down” represent 2 fold lower expression in the mutant. Fold change represents “average ”

^c^*P* value were determined by student t-test using Cyber-T .
