# Supplementary material for: Antibacterial Effects of Glycyrrhetinic Acid and Its Derivatives on Staphylococcus aureus
Source: PLoS One. 2016 Nov 7;11(11):e0165831. doi: 10.1371/journal.pone.0165831 (PMC5098735; doi:10.1371/journal.pone.0165831)
Supplement: S4 Table — (DOCX) [file pone.0165831.s005.docx]

S4 Table. Genes for amino acids metabolism up- and down-regulated by GR-SU

| Gene ID^a^ | Fold change^b^ | *P* value | characteristics |
| --- | --- | --- | --- |
| Down | | | |
| MW1856 | 0.25 | 0.0003 | hypothetical protein, similar to chorismate mutase/prephenate dehydratase (*pheA*) |
| MW1327 | 0.26 | 0.003 | threonine deaminase IlvA homolog |
| MW1328 | 0.27 | 0.005 | alanine dehydrogenase (*ald*) |
| MW1287 | 0.35 | 0.001 | hypothetical protein, similar to alanine racemase |
| MW0882 | 0.41 | 0.010 | thimet oligopeptidase homologue |
| MW1693 | 0.41 | 0.021 | D-alanine aminotransferase (*dat*) |
| MW0509 | 0.45 | 0.012 | branched-chain amino acid aminotroansferase homologue (*ilvE*) |
| MW1272 | 0.49 | 0.015 | hypothetical protein, similar to oligoendopeptidase |
| Up | | | |
| MW0414 | 2.54 | 0.004 | cysteine synthase homologue |
| MW0859 | 2.34 | 0.006 | hypothetical protein, similar to 2-isopropylmalate synthase |

^a^Based on the sequence of MW2 strain (accession no: NC_003923.1).

^b^”UP” represents GR-SU decreased the expression at more than 2 fold compared with that without treatment, while “Down” represent 2 fold lower expression in the mutant. Fold change represents “average ”.

^c^*P* value were determined by student t-test using Cyber-T
