# Supplementary material for: Antibacterial Effects of Glycyrrhetinic Acid and Its Derivatives on Staphylococcus aureus
Source: PLoS One. 2016 Nov 7;11(11):e0165831. doi: 10.1371/journal.pone.0165831 (PMC5098735; doi:10.1371/journal.pone.0165831)
Supplement: S5 Table — (DOCX) [file pone.0165831.s006.docx]

S5 Table . Genes for nucleic acids metabolism up- and down-regulated by GR-SU

| Gene ID^a^ | Fold change^b^ | *P* value | characteristics |
| --- | --- | --- | --- |
| Down | | | |
| MW0364 | 0.23 | 0.037 | xanthine phosphoribosyltransferase |
| MW0366 | 0.25 | 0.005 | inositol-monophosphate dehydrogenase |
| MW0694 | 0.26 | 0.020 | ribonucleoside-diphosphate reductase minor subunit |
| MW0693 | 0.30 | 0.030 | hypothetical protein, similar to comF operon protein 3 |
| MW0367 | 0.30 | 0.002 | GMP synthase(*quaA*) |
| MW0692 | 0.37 | 0.033 | NrdI protein involved in ribonucleotide reductase function |
| MW1286 | 0.42 | 0.019 | hippurate hydrolase |
| MW0450 | 0.44 | 0.002 | hypothetical protein, similar to 4-diphosphocytidyl-2-C-methyl-D-erythritol kinase |
| MW0837 | 0.47 | 0.012 | hypothetical protein, similar to polyribonucleotide nucleotidyltransferase |
| MW1157 | 0.49 | 0.023 | polyribonucleotide nucleotidyltransferase |
| MW1584 | 0.49 | 0.005 | GTP pyrophosphokinase |
| Up | | | |
| MW0110 | 3.74 | 0.0003 | purine nucleoside phosphorylase |

^a^Based on the sequence of MW2 strain (accession no: NC_003923.1).

^b^”UP” represents GR-SU decreased the expression at more than 2 fold compared with that without treatment, while “Down” represent 2 fold lower expression in the mutant. Fold change represents “average ”.

^c^*P* value were determined by student t-test using Cyber-T
