# Supplementary material for: Antibacterial Effects of Glycyrrhetinic Acid and Its Derivatives on Staphylococcus aureus
Source: PLoS One. 2016 Nov 7;11(11):e0165831. doi: 10.1371/journal.pone.0165831 (PMC5098735; doi:10.1371/journal.pone.0165831)
Supplement: S6 Table — (DOCX) [file pone.0165831.s007.docx]

S6 Table . Genes for transporters up- and down-regulated by GR-SU

| Gene ID^a^ | Fold change^b^ | *P* value | characteristics |
| --- | --- | --- | --- |
| Down | | | |
| MW1326 | 0.13 | 0.002 | hypothetical protein, similar to amino acid pearmease |
| MW0365 | 0.18 | 0.017 | xanthine permease (*pbuX*) |
| MW2325 | 0.30 | 0.022 | hypothetical protein, similar to NirC |
| MW0871 | 0.31 | 0.007 | oligopeptide transport system ATP-binding protein OppF homologue (*oppF*) |
| MW1325 | 0.38 | 0.009 | Blt-like protein |
| MW1220 | 0.38 | 0.010 | gamma-aminobutyrate permease (*gabP*) |
| MW0869 | 0.39 | 0.033 | hypothetical protein, similar to oligopeptide transport system permease protein OppC |
| MW1640 | 0.40 | 0.00009 | D-serine/D-alanine/glycine TRANSPORTER (*aapA*) |
| MW0870 | 0.40 | 0.010 | oligopeptide transport system ATP-binding protein OppD homologue (*oppD*) |
| MW0689 | 0.43 | 0.006 | hypothetical protein, similar to di-tripepride ABC transporter |
| MW1811 | 0.45 | 0.002 | hypothetical protein, similar to teichoic acid translocation ATP-binding protein *tagH* |
| MW0607 | 0.46 | 0.006 | hypothetical protein, similar to pyrimidine nucleoside transporter |
| MW1810 | 0.46 | 0.005 | hypothetical protein, similar to teichoic acid transport protein tagG |
| MW2287 | 0.48 | 0.034 | L-lactate permease *lctP* homolog |
| MW1776 | 0.49 | 0.028 | hypothetical protein, similar to ABC transporter *ecsB* |
| MW0163 | 0.49 | 0.035 | PTS enzyme II (EC 2.7.1.69), glucose-specific, factor IIA homologue |
| Up | | | |
| MW0698 | 2.22 | 0.011 | lipoprotein, similar to ferrichrome ABC transporter |
| MW2202 | 2.11 | 0.008 | hypothetical protein, similar to ferrichrome ABC transporter fhuD precursor |

^a^Based on the sequence of MW2 strain (accession no: NC_003923.1).

^b^”UP” represents GR-SU decreased the expression at more than 2 fold compared with that without treatment, while “Down” represent 2 fold lower expression in the mutant. Fold change represents “average ”.

^c^*P* value were determined by student t-test using Cyber-T
